# Impact of capacity building interventions on individual and organizational competency for HPSR in endemic disease control in Nigeria: a qualitative study

**DOI:** 10.1186/s13012-020-00987-z

**Published:** 2020-04-16

**Authors:** Obinna Onwujekwe, Chinyere Mbachu, Enyi Etiaba, Nkoli Ezumah, Uchenna Ezenwaka, Ifeyinwa Arize, Chinyere Okeke, Chikezie Nwankwor, Benjamin Uzochukwu

**Affiliations:** 1 Department of Health Administration and Management, University of Nigeria Enugu campus, Nsukka, Nigeria; 2 Health Policy Research Group, University of Nigeria Nsukka, Nsukka, Nigeria; 3 Department of Community Medicine, University of Nigeria Enugu campus, Nsukka, Nigeria

**Keywords:** Health policy and systems research, Getting research into policy and practice, Capacity building, Producers of evidence, Users of evidence

## Abstract

**Background:**

The need to build capacity for health policy and systems research (HPSR) in low- and middle-income countries has been underscored as this encompasses the processes of decision-making at all levels of the health system. This implementation research project was undertaken in Southeast Nigeria to evaluate whether the capacity-building intervention improves the capacity to produce and use research evidence for decision making in endemic disease control.

**Methods:**

Three training workshops were organized for purposively selected participants comprising “producers of evidence” such as health research scientists in three universities and “users of evidence” such as policy makers, program managers, and implementers in the public health sector. Participants also held step-down workshops in their organizations. The last workshop was used to facilitate the formation of knowledge networks comprising of both producers and users, which is a critical step for getting research into policy and practice (GRIPP). Three months after the workshops, a subset, 40, of workshop participants was selected for in-depth interviews. Information was collected on (i) perceptions of usefulness of capacity-building workshops, (ii) progress with proposed research and research uptake activities, (iii) effects of these activities on evidence-informed decision making, and (iv) constraints and enablers to implementation of proposed activities.

**Results:**

Most participants felt the workshops provided them with new competencies and skills in one or more of research priority setting, evidence generation, communication, and use for the control of endemic diseases. Participants were at different stages of planning and implementing their proposed research and research uptake activities, and were engaging across professional and disciplinary boundaries to ensure relevance and usefulness of outputs for decision making. Key enablers of successful implementation of activities were positive team dynamics, good balance of competencies, effective communication and engagement within teams, team leader’s capacity to innovate, and personal interests such as career progress. Lack of funding, limited decision space, organizational bureaucracies, and poor infrastructure were the key constraints to the implementation of proposed activities. Lack of mentorship and continuous support from trainers delayed progress with implementing proposed activities.

**Conclusions:**

The capacity-building interventions contributed to the development of a critical mass of research scientists, policy makers, and practitioners who have varying levels of competencies in HPSR for endemic disease control and would require further support in carrying out their medium and long-term goals.

Contributions to the literature

- Producers and users of HPSR evidence can be trained and empowered locally through capacity-building workshops. This has the potential to build a critical mass of context useful research scientists, policy/decision makers, and practitioners who know that successful endemic disease control programs rely on evidence-informed decision making and that health policy and systems research are viable tools for producing research evidence for endemic disease control.
- Embedding the formation of knowledge networks into capacity-building interventions for producers and users of research evidence enables continuous engagement across professional boundaries and enhances the possibility for future research collaborations for control of endemic tropical diseases.
- Knowledge networks of producers and users of evidence succeed in achieving set goals and objectives if leaders are committed and driven and if members of the network exhibit team spirit, communicate frequently (face-to-face or virtually), and receive on-going external support and mentorship from experts.

## Background

Policy makers, program managers, and implementers require a capacity to demand for and use research evidence for effective decision making that will achieve better health outcomes and reduce the burden of endemic tropical diseases. Such diseases continue to impose a tremendous health burden in resource-poor countries throughout the world, claiming millions of lives annually and inflicting severe morbidity that results in significant losses in economic productivity and social progress [1]. The most recent Demographic and Health Survey in Nigeria shows that the sustainable development goal (SDG) targets for malaria, and some other diseases are yet to be met and unlikely to be met by 2030 [2]. One of the reasons for this is a paucity of capacity in evidence-informed decision making, especially in the field of health policy and systems research (HPSR) and in getting research findings into policy and practice (GRIPP) [3–5].

The need to build capacity for HPSR in low- and middle-income countries has been underscored as this encompasses the processes of decision-making at all levels of the health system [6–8]. This activity falls within the realm of implementation science, which is the study of methods to promote the adoption and integration of evidence-based practices, interventions, and policies into routine health care and public health settings [9–11]. As both policy makers and communities increasingly demand better returns on investments in health, proper application of HPSR principles on policy making has the potential to enable health system interventions to achieve better value for money. HPSR enables the identification of gaps in capacity, barriers to efficient functioning, and effective performance of the health system and methods by which the existing resources can be optimally utilized [6, 12]. HPSR is typically context-specific and to apply research evidence to policy, capacity is needed at country level [13, 14]. The success of efforts to build capacity in developing countries in HPSR and other related areas will ultimately depend on political will and credibility, adequate financing, and a responsive research, capacity strengthening plan that is based on a thorough situational analysis of the resources needed for health research and the inequities and gaps in health care [15].

Research capacity encompasses the capacity to produce, demand for, and apply research, so that research evidence may contribute to improvements in health and health equity. However, reports show that the demand for research evidence is very low in LMICs [16, 17]. Various factors contribute to a lack of demand; there is a little appreciation of the value of research and its potential to contribute to policy development. “Another critical factor is that many LMICs do not have conducive environments or cultures for health research” [16]. Sustainable research capacity and evidence-informed policy making require health research professionals and policy makers with in-depth scientific expertise and complementary skills to enable implementation of independent, internationally recognized infectious disease research relevant to health priorities of their country. Strengthening the capacity of producers and users of research is arguably a more sustainable strategy for developing the field of HPSR in Africa than relying on training in high-income countries [6]. To achieve this, producers and users of HPSR evidence need to be trained and empowered locally in order to be more context useful. The long-term goal is to strengthen individual and institutional capacity to initiate and lead research activities in disease endemic countries, while developing national and international partnerships.

In Nigeria, universities are central to strengthening and sustaining the HPSR. They not only produce knowledge through research but are also mandated to teach the next generation of policy makers, health professionals, and researchers [7]. However, there is limited capacity among these groups due to the long-standing culture of not making research a priority and poor funding for research [1, 3]. Recognizing the increasing need to build capacity in HPSR in both the “pull and push” domains of research, and to develop context-specific strategies for control of neglected tropical diseases (NTDs) and malaria in Nigeria, capacity-building interventions were organized for relevant stakeholders in two Nigerian states. The specific objectives of the interventions were to determine the needs of producers and users of evidence in priority setting for HPSR in the control of endemic diseases; introduce them to the field of HPSR, the concept of GRIPP, and economic evaluation; and facilitate their application of these concepts in planning and implementing research and research uptake activities for the control of NTDs and malaria in their respective states. These interventions consisted of training workshops that were targeted at “producers” and “users” of research evidence and facilitated by researchers from Health Policy Research Group, College of Medicine University of Nigeria [7].

This paper reports findings from the evaluation of the impact of capacity-building interventions on individual competence and organizational capacity to implement research and research uptake activities for control of endemic tropical diseases. It also highlights key contextual enablers and constraints to the implementation of short-term research and research uptake activities for control of endemic tropical diseases.

### Description of capacity-building intervention

The capacity-building intervention was preceded by a HPSR, and GRIPP capacity needs assessment of policy makers, practitioners, and research scientists in Anambra and Enugu states. The study participants consisted of 118 purposively selected respondents who were currently or previously involved in endemic tropical disease research or programming in the study states. Data was collected data using two different questionnaires for the two categories of respondents (producers and users of research evidence). The questionnaire for producers of evidence elicited information on their involvement in HPSR+A, communication of research findings, and engagement with policy makers for knowledge translation. The questionnaire for users of evidence elicited information on patterns of use of evidence for policy and decision making, demand for and capacity to initiate research, and enablers and constraints to evidence-based decision making. The assessment revealed gaps in the capacity to produce and use evidence for decision making in control of endemic diseases in both states, and a need to build a critical mass of users and producers of evidence in HPSR+A for better control of endemic diseases. Findings from the needs’ assessment have been published elsewhere [18].

Following the capacity needs’ assessment, three workshops were organized to train participants on HPSR, GRIPP, and economic evaluation for endemic disease control. The first workshop focused on HPSR and economic evaluation, the second was on GRIPP, and the third was a step-down workshop on HPSR, economic evaluation, and GRIPP.

In the first and second workshops, producers and users of evidence were trained together, whereas parallel sessions of step-down training were adopted for the third workshop. Each workshop lasted for 2 days. A total of 118 people comprising 54 producers of evidence and 64 users of evidence attended the workshops. Table 1 highlights numbers and categories of participants who attended each workshop. All users of evidence who attended the first workshop also attended the second workshop. However, there were 4 producers of evidence who could only attend the first workshop due to conflicting schedules.

**Table 1:** Capacity-building workshops attended by survey participants

Training workshops organized by the Health Policy Research Group	Modules/topics covered	Frequency (%)	
		Producers of evidence (*N* = 54)	Users of evidence (*N* = 64)
Workshop 1: Training of trainers on health policy and systems research & economic evaluation	• **HPSR** Introduction to complex health systems Introduction to health policy and health system governance Introduction to health sector reform Conducting literature review Health policy and stakeholder analyses Health systems research priority setting • **Economic evaluation** Introduction to health economics and health technology assessment Pharmaco-economics and outcomes research for disease control Application of cost effectiveness analysis and health technology assessment for decision making	23 (42.6)	24 (37.5)
Workshop 2: Training of trainers on getting research into policy and practice	• **GRIPP** Monitoring and evaluation of health programs Knowledge networks for health research GRIPP: principles, methodologies, and benefits Advocacy for GRIPP Leadership for health research Managing political and socio-cultural interferences in policy making Entrenching research	19 (35.2)	24 (37.5)
Workshop 3: Step-down training on HPSR, economic evaluation, and GRIPP	Modules in HPSR, economic evaluation, and GRIPP (as above)	39 (72.2)	48 (75)

On the last day of the third workshop, a combined session for producers and users of evidence was held, during which participants were grouped into four thematic knowledge networks of NTDs, malaria, maternal and child health, and health system strengthening. Participants were assigned to groups based on their previous area of work or interests, or their current work or interest.

Each thematic network, comprising both producers and users of evidence, was asked to brainstorm on research and research uptake activities they would commit to undertake within 3 months of the training workshop in order to contribute to evidence-informed decision making. As a follow-up activity, the participants were encouraged to hold step-down trainings for colleagues in their organizations to help increase the numbers of producers and users of evidence with the requisite knowledge of HPSR and GRIPP. The formation of knowledge networks of producers and users of evidence was considered a critical first step for knowledge translation and GRIPP. The network of producers and users of evidence was supposed to provide a platform for research evidence to be interpreted in a manner in which policy makers and practitioners would understand and find useful for policy and practice.

The first two workshops (training of trainers) were led by experts in HPSR, Health Economics, and GRIPP. The experts included 3 professors of health economics, health systems and policy, and sociology and social determinants of health, and 3 assistant professors (senior lecturers) of health economics, health systems and policy, and public health. Each thematic group was led by a professor in the area, with considerable practical experiences in implementation science. They were assisted by middle-level academics that also had the expertise in the various thematic areas. The third workshop (step-down training) was facilitated by trained producers and users of evidence, with the assistance of experts in HPSR, Health Economics, and GRIPP. The modules covered in each training are shown in Table 1. The agenda and materials that were used for the intervention are available from the corresponding author on request.

After a waiting period of 3 months, participants’ progress in implementing proposed short-term activities was evaluated to assess whether and how the capacity-building intervention had improved their capacity to produce and use research evidence for decision making in endemic disease control. Since there is no guideline on the ideal interval between the implementation of interventions and assessment of the effects of the interventions, 3 months were considered a reasonable interval. In addition, the high level of mobility of research users (policy makers and practitioners) resulting from transfers to other duty positions necessitated a relatively short waiting period. Although evaluating after a short interval runs the risk of having participants still being at the planning stage of implementation, we considered it a better option to evaluate in 3 months rather than wait longer and run the risk of loss to attrition of most people whose capacity were built.

## Methods

### Study aim

The aim of the study was to evaluate the effect of the capacity development interventions on individual competencies and organizational capacity to implement proposed HPSR and GRIPP activities for the control of endemic diseases in Anambra and Enugu states, southeast Nigeria. The study also explored contextual factors influencing the implementation of proposed activities for evidence-informed decision making.

### Study design

Qualitative research approach was used to collect information from purposively selected respondents (details provided subsequently, see the “Participant selection” section) who were either producers of evidence (researchers and academia) or users of evidence (policy makers, program/project managers) in Enugu and Anambra states.

### Study setting

The study was implemented in Anambra and Enugu states. Both states are located in southeast geopolitical region of Nigeria. As with other regions in Nigeria, the prevalence of malaria is high, and the disease burden is highest among pregnant women and children under 5 years of age (15.2% in Anambra and 30.2% in Enugu) [2]. Similarly, the prevalence of NTDs is high in the region. At the time of the study, both states were receiving funding support through Saving One Million Lives to expand access to essential primary health care services (including malaria control) for women and children. The initiative is focused on evidence-based decision making to address the leading causes of morbidity and mortality in the country.

### Participant selection

A subset (40) of workshop participants was invited to participate in key informant in-depth interviews. Respondents were purposively selected based on (i) potential or actual roles in producing and using research evidence for decision making in endemic disease control and (ii) contributions to knowledge networks. This included researchers, lecturers, policy makers, program/project managers, and senior healthcare managers.

Producers of evidence were drawn from government-owned tertiary institutions including the universities and teaching hospitals in both states. Users of evidence were drawn from relevant state ministries/departments/agencies, local government health departments, and disease control programs.

The participants were purposively selected so that the critically relevant users of evidence from research (policy makers and program managers) and producers of evidence (researchers) were recruited for the study. The policy makers and program managers included top and mid-level managers from the Ministries of Health in both states. These are people who are directly involved in policymaking, program planning, and implementation. A list of the potential participants was first drawn, then the research team and permanent secretaries in both Ministries selected participants bearing in mind the maximum number of possible participants the project can accommodate. Unavailable and unwilling participants were replaced by a deputy or someone who plays a similar role in their organization. Healthcare managers who are not directly involved in decision making were excluded from the training. In the case of researchers, the research team selected mid-career and senior HPSR+A researchers from public universities in the two states, based on the team’s knowledge of researchers’ capacities. The selection was done to ensure gender balance and to include at least ten prolific researchers in each state. We defined prolific researchers as those who have published at least 5 manuscripts in the subject area or a related area. These people were approached, sensitized about the project, and recruited to be part of the project.

### Data collection and analysis

Data was collected using pre-tested semi-structured interview guides that were first pre-tested to ensure content and construct validity (see Additional file 1). All interviews were conducted by male and female experienced qualitative researchers and/or lecturers. Each interview was conducted privately in respondent’s workplace by a team comprising an interviewer and a note-taker. All interviews were audio-recorded with the permission of respondents, and each lasted for 45–60 min. Audio files were transferred to password secure laptops and encrypted. Handwritten notes were typed in word and linked to corresponding audio files. Interviewers met weekly to debrief.

The purpose of the interviews was to achieve coverage and representation rather than the saturation of information, that is to interview as many workshop participants as possible to ensure data is collected from each thematic network/group of workshop participants for both users and producers of evidence. Audio files were transcribed verbatim and transcripts were coded manually by six coders based on pre-identified themes (from the study aims/objectives) and those that emerged during the initial coding of selected transcripts. Four rich transcripts (one from each category of the respondent from each state) were selected and manually coded to test the pre-identified themes and identify emerging themes. The coding framework was then agreed on by the entire research team and applied to all 40 transcripts. The major themes include (i) individual competency gained in HPSR, (ii) organizational capacity to use evidence for policy/decision making and practice, (iii) process of planning and implementation of proposed activities on HPSR, and (v) contextual influences on the use of evidence for decision making. Discrepancies among coders were resolved by consensus; there was no need to involve a third coder. Synthesized findings were endorsed by participants during a validation meeting.

## Results

Forty in-depth interviews were conducted among the two categories of study participants in both states. The characteristics of key informants for in-depth interviews are shown in Table 2.

**Table 2:** Characteristics of key informants for in-depth interviews

Respondent code	Gender	Role	Respondent code	Gender	Role
Producers of evidence			Users of evidence		
**Anambra state**					
AP01	Female	Principal pharmacist	AU 01	Male	Policy maker
AP02	Female	Resident doctor	AU 02	Male	Program manager
AP03	Female	Lecturer (nursing)	AU 03	Male	Policy maker
AP04	Male	Lecturer (pharmacy)	AU 04	Male	Policy maker
AP05	Male	Medical practitioner	AU 05	Female	Program manager
AP06	Female	Assistant director of pharmacy	AU 06	Male	Program manager
AP07	Female	Chief medical Laboratory scientist	AU 07	Male	Program manager
AP08	Female	Lecturer (nursing)	AU 08	Female	Policy maker
AP09	Female	Professor of public health	AU 09	Female	Program manager
AP10	Male	Professor of public health	AU 10	Male	Program analyst
**Enugu state**					
EP01	Female	Academician	EU01	Male	Program manager
EP02	Male	Practitioner	EU02	Female	Program manager
EP03	Male	Practitioner	EU03	Female	Program manager
EP04	Male	Academician	EU04	Female	Program manager
EP05	Male	Practitioner	EU05	Male	Policy maker
EP06	Male	Practitioner	EU06	Male	Program officer
EP07	Female	Academician	EU07	Male	Policy maker
EP08	Female	Practitioner	EU08	Male	Program manager
EP09	Male	Practitioner	EU09	Male	Program manager
EP10	Male	Practitioner	EU10	Male	Policy maker

### Knowledge and skills gained from capacity-building intervention

Respondents highlighted they had gained individual competencies in HPSR and GRIPP, some of which they had been able to share with colleagues and students. The perception of types and amount of competency gained through the workshops varied among respondents. At organizational levels, participation in the workshop was perceived to strengthen the capacity to use research evidence for program planning and management, as well as budgeting for health programs to ensure efficiency in resource allocation and use. Feedback about changes at the organizational level was provided by the unit and departmental heads who reported that the workshop had contributed to improvements in evidence use for planning and programming in their departments or units.

#### Individual competencies in HPSR evidence generation, communication, and use for the control of endemic diseases

Various respondents reported that as a result of the capacity-building workshop, they have a better appreciation for the usefulness of evidence in policy making, are more competent to undertake HPSR and economic evaluation of endemic disease programs, and are better able to communicate research evidence to health policy makers and program managers.

It has heightened my interest in health policy issues and influenced my choice of topics. When choosing a topic, I go for topics looking at problems we have on ground and topics that will address those health policy problems. (AP02; Medical practitioner)

What I gathered is that policy should be evidence-based. I have come to realize that before a policy is made, we have to first go into research to find out the needs of the populace so as to be able to advise the policy makers to make the right policy (AP01; pharmacist).

We collaborate with the malaria control program officer at the ministry so whatever information we have, whatever data we have, we share with them (EP05; Medical practitioner).

Some respondents highlighted that participating in the workshops broadened their research interests, enabled them to realize possibilities beyond their field of practice, and helped them focus their research.

...as a social scientist, the knowledge I got from the program, on maternal mortality in Nigeria and the issues about child care really broadened my interest, and made me want to do some research on MCH (EPO1; academician).

I’ve been trying to focus on my PhD research ...after the program I decided to pick a topic ...that will impact on policy [and] be meaningful to the society (EPO7; academician)

The team that proposed to revive the research component of the Department of Planning, Research and Statistics in Enugu State reported that they were able to effectively communicate to high-level decision makers in the ministry about the need to revive the unit and recommended strategies that could be applied. A health information management officer was able to properly monitor and report data collected from health interventions using the knowledge and skills he acquired during the workshop.

Policymakers need us to tell them what to do to have a viable research unit. So we developed strategies [recommending that] the unit [needs] adequately furnished office and necessary gadgets to work with; that there is need to train public health officers on research methods and...the need to employ a health economist to be part of the budget planning (AU01; policymaker).

The monitoring we embarked on about two weeks ago, the skills I applied in report writing and the rest of them which I have submitted to the management was basically from the workshop (EU06; program manager).

Few respondents could not clearly identify or demonstrate new skills acquired from the workshop.

#### Individual competencies in HPSR evidence use for planning and implementation of endemic disease control interventions

Respondents gained various competencies in planning and implementation of endemic disease control programs and were able to apply knowledge and skills acquired in (i) daily decision making; (ii) supervision and monitoring of malaria control program; (iii) research networking for malaria; (iv) data collection, analysis, reporting, and use for planning immunization and other disease control programs; and (v) budgeting for activities using data obtained from program evaluation.

In Anambra state, a program manager was able to plan, cost, and budget for health interventions in NTDs using the knowledge and skills acquired during the workshop.

The workshop really helped me a lot in NTDs program especially as it regards health economics because you just don’t go into the field. You have to sit down and do the costing and planning and this has really helped us in NTD programme (AU02; program manager).

In Enugu state, there were reports of improved capacity to monitor projects, influence individual practice, and apply evidence in every day activity.

The workshops also enhanced data collection, reporting, and use. A health information management officer was able to properly monitor and report data from health interventions using the skills he acquired from the workshops. Another respondent highlighted that participating in the workshops created in him a consciousness to always use quality data/evidence for decision making.

#### Some respondents had begun sharing knowledge gained or advocating for and influencing colleagues to use research evidence for decision making

Some respondents reported that they had embarked on some form of knowledge transfer, sharing what they learnt from the workshops with their colleagues and/or students, and advocating for policy-relevant research.

Incidentally, my friend is doing her PhD on compliance to maternal and child health care services in Anambra state. So, I told her that rather than looking [from a generic perspective] she should [disaggregate] to help her create a model (EP01; academician).

In Anambra state, a lecturer reported that she now emphasizes the importance of debunking myths and misconceptions about communicable diseases, especially neglected tropical diseases, and the need to seek appropriate treatment, while a program manager said the workshop motivated him to sensitize colleagues and advocate for evidence-based decision making.

Among producers of evidence in Enugu state, although there were reports that the workshops had built the capacity of staff and enabled knowledge transfer, there was an erroneous perception that evidence-informed decision making is a responsibility of policy makers and program managers in the Ministry of Health.

Well, you know the use of evidence for decision making is mainly the duty of those at the ministry. They are the implementers, those are the policy makers, program managers.... we are not generating much data here yet for them (EP05; medical practitioner).

#### Organizational capacity to use evidence for policy/decision making and practice also improved

Several respondents stated that their institution has applied the skills acquired in the following ways: (i) design of annual operational plans; (ii) planning and costing of NTD programs such as preventive chemotherapy for NTDs and innovative disease management of NTDs in the state; (iii) planning and decision making, at the advisory and management levels, for saving one million lives program to achieve greater value for money; and (iv) budgeting for maternal and child health programs and interventions.

My organization has applied the skill acquired in so many ways; in budgeting for our maternal and child health week and also building capacity of users during a program on integrated management of childhood illnesses (AU08; policy maker).

In Enugu state, respondents perceived improvements in organizational capacity to use evidence in decision making and practice, especially in planning for activities and budgeting. This was enhanced by on-going knowledge transfer activities and inter- and intra-departmental collaborations. Other organizational capacity improvements include greater ability to innovate around bottlenecks using forums established during the workshop, which enabled members to make contributions during management sessions and plan for continuous monitoring of proposed activities to facilitate evidence-informed decision making.

### Progress with the implementation of proposed activities by producers and users of evidence

Information on participants’ progress with the implementation of proposed HPSR and GRIPP activities was collected to provide a more tangible assessment of capacity improvements following training workshops. These findings are structured according to cross-cutting activities (Table 3), and according to thematic working groups (Table 4), and each activity is further analyzed for its potential to contribute to individual competency and/or organizational capacity to undertake HPSR and GRIPP.

**Table 3:** Progress with the implementation of proposed cross-cutting activities

States	Proposed activities	Progress with proposed activities	Purpose of activity (influence on the capacity for HPSR and GRIPP)
Anambra	Advocacy to Commissioner and Permanent Secretary of Health for evidence-informed decision making	Visited both policy makers and informed them of potential strategies to strengthen evidence-based decision making for endemic disease control	To improve organizational capacity for GRIPP
	Reactivation of the Department of Planning, Research and Statistics for promoting research evidence generation and use	Drafted and disseminated (to the relevant authority in the Ministry of Health) proposals with strategies for reactivating the unit	To improve organizational capacity to undertake HPSR and GRIPP
	Advocacy to the relevant authority for research funding in the Ministry of Health	Have advocated to relevant sectors and are discussing research funding partnerships with implementing partners and private sector	To improve organizational capacity to undertake HPSR
Enugu	Reactivation of research unit within the Department for Planning, Research and Statistics for promoting research evidence generation and use	Scheduled agenda-setting meetings Undertook stakeholder analysis Identified action points and assigned tasks to members	To improve organizational capacity to undertake HPSR and GRIPP

**Table 4:** Progress with the implementation of proposed theme-based activities

Thematic network	Proposed activity	Progress with implementation	Purpose of activity (influence on capacity for HPSR and GRIPP)
Neglected tropical diseases			
Anambra	Review existing literature on the prevalence of NTDs	• Carried out a scoping review of epidemiological studies on NTDs • Held series of meetings to compare individual findings towards data synthesis	To improve individual competence and organizational capacity to undertake HPSR
	Capacity building of local government NTD focal persons and other users of evidence in the state on HPSR	• Leveraged on an on-going program to step-down training workshop on HPSR to NTD focal persons at local government level	
	Advocate for evidence-based decision making	Planning for implementation	To improve organizational capacity for GRIPP
Enugu	Mobilize and sensitize key stakeholders and community leaders	• Sensitized community on NTDs and need for increased uptake of free Ivermectin for onchocerciasis	To improve program implementation
**Malaria**			
Anambra	Undertake survey on the availability of malaria diagnostic tools and personnel in selected local government areas in the state and disseminate findings to policy makers	• Conducted a pilot study on the utilization of anti-infective medicine • Mapped out study area • Begun reviewing literature and designing the study protocol	To improve individual competence in HPSR
	Research priority setting in malaria	• Data collation to enable identification of research priorities	To improve organizational capacity for GRIPP
Enugu	Evaluate the distribution of LLINs and effectiveness of ACTs	Nothing to report	
**Maternal and child health**			
Anambra	Assessment of utilization of maternal health services	• Completed literature reviews on the proposed research topic • Developed study protocol and obtained ethical approval • Met with health workers in the proposed study site	To improve individual competence in HPSR
Enugu	Periodic survey on access to free MCH	Nothing to report	
**Health systems strengthening**			
Anambra	Evaluate the effect of training various cadres of health workers on timely/prompt patient care to reduce hospital waiting time	• Process of collecting data for a baseline study (on ways of improving patient care) which will inform the design of training intervention for health workers	To improve individual competence in HPSR
Enugu	Assessment of clients’ perceptions of service delivery in tertiary hospitals	• Team meetings to discuss findings from the literature review and brainstorm on research design	

#### Thematic groups of producers and users of evidence were at various stages in planning and implementation of proposed theme-based and cross-cutting activities

Table 3 shows that participants had begun implementing all proposed cross-cutting activities and the purpose of each activity is to improve organizational capacity for HPSR and/or GRIPP.

With the exception of malaria and MCH thematic groups in Enugu state, participants had begun implementing proposed theme-based activities. Table 4 shows that most of these activities aim to improve individual competence for HPSR and a few aim to improve organizational capacity for HPSR and GRIPP.

### Contextual influences on implementation of proposed activities

**Fig. 1:**
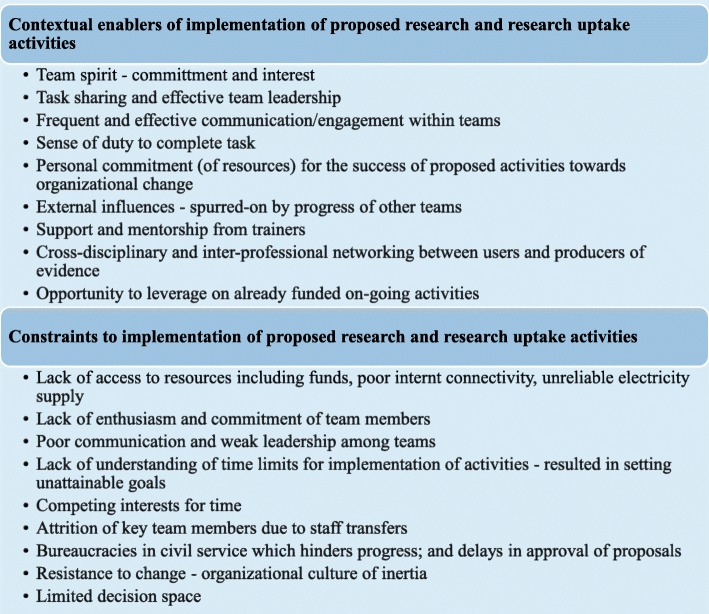
Contextual enablers and constraints to implementation of proposed theme-based and cross-cutting activities (Source: Health Policy Research Group. 2015. Capacity building of producers and users of evidence in HPSR for control of endemic tropical diseases. Technical report. Enugu, Nigeria)

Several factors facilitated or constrained the implementation of research and research uptake activities proposed by thematic knowledge network groups. These factors are highlighted in Fig. 1.

Key enablers of successful implementation of activities were political support, positive team dynamics, good balance of competencies, effective communication and engagement within teams, team leader’s capacity to innovate, and personal interests such as career progress.

The major thing that helped was the interest, most of the people are interested, and the knowledge is there for us to do it (EP10; medical practitioner)

We are enjoying the audience and support of our Hon. Commissioner of Health; also my team members are committed and supportive (AU01; policy maker)

We had series of meetings and then we also have this WhatsApp group where we communicate regularly. (AP03; lecturer, nursing dept.)

Lack of funding, limited decision space, organizational bureaucracies, and poor infrastructure were key constraints to implementation of proposed activities. Lack of mentorship and continuous support from trainers delayed progress with implementing proposed activities.

[We do not have] appropriate authority for us to execute our programs and again lack of funds (AU 10; programme analyst)

…the major challenge we had was time factor and again lack of finance to carry out these activities (AU09; programme manager)

Time is a factor since all of us work full time in our respective places of work. And then we have very poor internet, you know ICT accessibility and availability, since each person in the group each member of the group depends on himself or herself to fund the cost of the ICT he or she uses. So funding is a very strong constraint indeed and then time. (AP09; professor of public health)

The leader of the group and the rapporteur collected email addresses and phone numbers of people, and agreed to arrange a WhatsApp group [chat] to enable us to carry out those things we agreed to do. But up till this very moment, the WhatsApp group has not been created, neither has any mail been forwarded to that effect (EP02; medical practitioner)

A program manager in Enugu state specifically highlighted that lack of (political) support from management (referring to key decision makers in the Ministry of Health) made it impossible to reactivate the research unit as proposed.

We have the willingness, but we don’t have support from our management. We cannot actually roll it [proposed activity] out. They [referring to management] have already been sensitized on the need to give research a priority, just that they are not putting it into practice…. they have not really addressed the issue of research… (EU03; programme manager)

## Discussion

This was a 12-month implementation research project to build the capacity of producers and users of evidence in generation and use of HPSR findings to improve control of NTDs and malaria, primarily. Although this project was carried out in two states, the intent was not to have compared between states or between producers and users of evidence, rather to build a critical mass of research scientists, policy/decision makers, and practitioners who know that successful endemic disease control programs rely on evidence-informed decision making, and that HPSR and economic evaluation are viable tools for producing research evidence and commit to undertake activities towards improving the use of research evidence in endemic disease control. An intended consequence of the project was to build networks between producers and users of evidence and hopefully begin to bridge the gap between them to facilitate and enhance GRIPP [5]. Some of the outputs and findings from this study can be found in a WHO proposed framework for GRIPP for different health systems [19]. This evaluation also highlighted some enablers and constraints to participants’ capacity to implement proposed GRIPP activities which have also been elaborated in similar studies [20, 21].

The fact that participants felt that the workshops improved their competencies and skills in research priority setting, evidence generation, and communication not just for the control of endemic diseases but for the holistic evidence-based improvements of the health system shows the potential for increasing evidence-informed decision making with appropriate interventions as shown in previous studies [22–25]. This is a very timely project in Nigeria and some other African countries, where it has been found that the capacity for research and knowledge translation activities, although growing, still remains largely uncoordinated and remains as small-scale activities, and primarily driven from outside Africa [26]. In this study, continuous knowledge transfers and engagement between knowledge networks of policy makers and researchers were documented. It has been proven that the interpersonal relationship between policy makers and researchers is a better approach of strengthening their collaboration and bridging the gap between them [23]. This study provided a platform for interaction among experts (researchers and policy makers) in different sectors, thus enhancing the possibility of future collaboration, and this has been found to be very essential in implementation research promotion [27]. Studies and reports have shown that frequent interaction between researchers and policy makers will promote the implementation of evidence-based policy [28–32].

Another study carried out in a different state in Nigeria, recognized that strategies employed in our study for evidence-based policy making and implementation are likely to produce better outcomes in developing countries like Nigeria [3]. It also suggests a supply-driven approach to capacity-strengthening initiatives based on the assumption that if the skills of the main actors (researchers and policy makers) are enhanced via trainings, and institutional capacity is built, research outputs will be put to good use [3]. An example is the use of the Health Policy Advisory Committee which comprises policy makers and researchers, as well as other stakeholders in the health sector. This serves as an excellent mechanism to bridge the divide between producers and users of evidence and a good platform to promote intersectoral partnership, collaboration, and networking to facilitate evidence-to-policy linkage [33].

Knowledge translation platform was embedded in the design of this study and found to be useful in complementing capacity-building activities and influencing initiatives for evidence-informed health system policy-making [34, 35]. There is a need to further develop knowledge translation platforms to strengthen health systems as the scarcity of evidence exists on the influence of knowledge translation platforms, especially in low- and middle-income countries [31]. This will contribute towards learning the health systems which is stated as most needed to achieving the sustainable development goals [36].

The short duration of this project and the preliminary findings from the evaluation of participants’ short-term goals reveal that they will require further support in carrying out their medium and long-term goals. Evidence has shown that mechanisms like the secondary assignment of researchers in top policy making positions can promote and influence evidence-informed policy-making [33]. However, secondary assignments require substantial investment, with emphasis on high technical positions to ensure GRIPP [33, 37].

Although respondents were better equipped with skills to undertake HPSR and communicate findings to policy makers, their competencies could not be objectively assessed because they were either yet to implement or complete proposed short-term activities and produce results for dissemination. The use of a self-assessment technique to assess training outcomes is prone to bias and is a major limitation of this study [38]. However, this was reduced by individual in-depth interviews with a considerable number of respondents. Also, the fact that the full demographic information of the participants apart from gender and role in the organization was not obtained during qualitative interviews with participants is a study limitation because it possibly limits the inferences of some of the relationships of individual demographics with outcomes. This will be taken into consideration in future studies. An additional weakness of this study includes a lack of preliminary data which limits the ability to infer that the intervention achieved desired goals. Also, participants were selected on purpose and organizational capacity was not captured outside of their perceptions.

## Conclusions

Capacity-building interventions are successful in improving individual and organizational capacity for HPSR in endemic disease control. Given the short duration of this project, preliminary findings from the evaluation of participants’ short-term goals reveal that they will require further support in carrying out their medium and long-term goals. The main cross-cutting constraints were time and funding and, for some others, the additional constraint of limited decision space/authority to make GRIPP changes. These issues have been identified by some capacity assessment studies. Recommendations include continuing sensitization, support with advocacy, expanding critical mass of producers and users of HPSR evidence, and building bridging networks.

## Supplementary information

**Additional file 1.** In-depth interview guides for producers and users of evidence.

## Data Availability

Some data generated or analyzed during this study are included in this published article. Additional data (generated and analyzed) are available from the corresponding author on reasonable request.

## Abbreviations

ETD: Endemic tropical disease
GRIPP: Getting research into policy and practice
HPSR: Health policy and systems research
IDI: In-depth interviews
LMIC: Low- and middle-income countries
NTD: Neglected tropical diseases
SDG: Sustainable development goals
WHO: World Health Organization
