# Supplementary material for: Impact of capacity building interventions on individual and organizational competency for HPSR in endemic disease control in Nigeria: a qualitative study
Source: Implement Sci. 2020 Apr 16;15:22. doi: 10.1186/s13012-020-00987-z (PMC7164165; doi:10.1186/s13012-020-00987-z)
Supplement: Supplementary file 1 — Additional file 1. In-depth interview guides for producers and users of evidence. [file 13012_2020_987_MOESM1_ESM.docx]

**Building Capacity of Users and Producers of Evidence in Health Policy and Systems Research for Better Control of Endemic Diseases in Nigeria**

**Tools for Next Steps Evaluation Activities for Producers and Users of Evidence in Anambra and Enugu States**

**Information and Consent**

**Introduction:**

Good morning/afternoon. My name is ________. Following your participation in HPRG’s capacity building workshop(s), and your subsequent inclusion in one of the thematic knowledge network groups; viz, NTDs, MCH, Malaria and HSS, we are here to conduct an assessment which will help us evaluate how our capacity building efforts have positively (or negatively) affected participants, and to know what these efforts have yielded in wider organizational contexts in terms of evidence-based decision making, cultural or organizational change, knowledge management and relationship building. Please, feel free to seek clarifications from me if there are any issues you do not understand along the line.

With your consent, interviews will be audio-taped. Only the research team will have access to the tapes which will be coded with identification numbers to protect your identity. The tapes will be destroyed 12 months after the project. You will also receive a feedback on the findings at the end of the project.

**Possible risks and benefits**

We do not foresee any risks that could result from taking part in this study**.** You will have no personal benefit from participating in this interview, but the findings and outputs from this project will contribute to improving the control of endemic tropical diseases and could influence future policy making in this field in Nigeria.

**If You Decide Not to Be in the Study**

Participation in this study is voluntary which means you are free to decide if you want to be in it or not. If you decide not to participate, there will be no negative implications for you or your work. You may skip the questions you are not willing to answer.

**Confidentiality**

We will, to the best of our ability, protect information about you and your participation in this study. We will not use your name in any reports or publications. The transcripts from the study will only be handled by the research team.

**DECLARATION: TO BE SIGNED BY THE RESPONDENT GIVING CONSENT**

**Agreement of respondent:**

**The purpose of the interview was explained to me and I agree to participate.**

**____________ ______________________________**

**Signed Date**

**IDI Guide for Evaluation of Proposed GRIPP and HPSR Activities in Enugu State - Users of Evidence**

| **Questions in activity** | **Prompts** |
| --- | --- |
| **NTDs**   1. Of what relevance was the conduct of a situational analysis of intensified disease management (IDM) in Enugu state? What were your findings? 2. How did you carry out a review of community directed distributors (CDD) data and activities in Enugu state and what was the outcome? 3. How did you mobilize and sensitize stakeholders (including town union presidents and other opinion leaders)? What was achieved? 4. What benefits did you receive from your advocacy visits to decision makers; viz commissioners of health, budget & planning, local government etc? 5. In what ways have these activities affected use of evidence for decision making (influence on individual practice and organizational culture) in the control of malaria and other NTDs   **Malaria**   1. What were your findings in evaluation of distribution of LLINs in Enugu state? 2. What were your findings in evaluation of effectiveness of ACTs use in Enugu state? 3. What knowledge translation methods did you engage with colleagues? Were they useful and did they produce any palpable results? 4. How did you step-down HPSR modules to malaria unit members in all levels of the SMoH? Which categories were chosen and why?   **MCH**   1. How did your advocacy visits to women groups to encourage their pro-active decision making on MCH turn out? Did they welcome the idea? How can the idea be propagated better? 2. How can community leaders help sensitize their communities on identifying danger signs in MCH? Did you approach any community leaders? 3. Were there any advocacy campaigns designed by you (and group members) to include breast examination as part of ante-natal routine for early breast cancer detection? If yes, did you already advocate to any individuals, group or organisation? 4. How has your knowledge network {Free Maternal & Child Health (FMCH) Network} improved wider organisational strategy and change? 5. How can periodic (quarterly) survey in collaboration with the programme management unit of FMCH in the SMoH contribute to practice change? Did you achieve any collaboration with the programme management of FMCH? Did you pay advocacy visits to the managers of the programme? 6. What roles do traditional rulers, town union presidents and other stakeholders have in the FMCH? Did you pay any advocacy visits to any stakeholders? If yes, what was the outcome?   **HSS**   1. What research group was formed? What has been done so far by the group? 2. Have you conducted an evaluation on a tracer service delivery component? What are the findings? How were you able to support researchers and research centres? 3. Were there monthly data review meetings by your group? Did you address data governance issues | ****Ask reason(s) if activity not executed***  *(Probe for group planning and approach, methodology and lessons learnt from exercise)*  *(Probe for planning process methods used, expected and actual outcome)*  *(Probe for who, how, where and outcome of the advocacy*  ****Ask reason(s) if activity not executed***  *(Probe for group planning and approach, methodology and lessons learnt from exercise)*   - *(Explore knowledge of methods and range and levels of colleagues engaged)*   ****Ask reason(s) if activity not executed***   - *(Probe for which women groups, what was done, and outcome)*   *(Probe for which communities, types of community leaders)*  *(Probe for planning process methods used, expected and actual outcome)*  *Explore perceptions of the effect on organisational management*  *(Explore effects of collaboration?)*  *(Probe for which communities were visited)*  ****Ask reason(s) if activity not executed***  *Probe for when and how*  *(Probe for planning process methods used, expected and actual outcome)* |

**IDI Guide for Evaluation of Proposed GRIPP and HPSR Activities in Enugu State - Producers of Evidence**

| **Questions in activity** *(*Note that all proposed respondents are to attempt all question irrespective of their thematic area)* | **Prompts** |
| --- | --- |
| **General**   1. Do you think this program has built your capacity to carry out research in the areas of Health policy and systems? 2. How has the knowledge from the workshop helped you enhance the control of endemic tropical diseases such as malaria and NTDs in Southeast Nigeria? 3. How did the workshop help you with economic evaluation and equity analysis, in order to enhance the contribution of HPSR+A in improved control of endemic diseases? 4. Have you met as a group after the workshop? What activities did you do in the meeting? 5. Did you engage/collaborate with the **users of evidence** since the last workshop? What was the engagement for and what was the outcome?   **NTDs**   1. Which laboratories are used for the investigations to confirm the diagnosis of NTDs in the State? Are they well known to the health workers in the state? 2. Were there talks/moves to sensitize the government on the need to get more laboratories? 3. Did you carry out any advocacy visit to help form a research group? 4. If yes, give details of what was done. 5. Are there plans of alternative sources of funding for the research or treatment of people with NTDs in the State? 6. Did you and your group make any effort to create any awareness about NTDs to people in the State? Explain in details what was done. 7. Are there plans to carry out epidemiological mapping of NTDs in the State?   **Malaria**   1. Was there any analysis carried out to evaluate the strengths, weaknesses, opportunities and threats to the current malaria program in the state? 2. How do you intend to disseminate the information gotten about the malaria program to the users of evidence, policy makers and the general public? 3. Have you planned on how to structure the information management system in the state to capture adequate malaria information to help inform decisions that will affect the elimination of malaria in the state?   **HSS**   1. Have you met to form a research group that will undertake the evaluation of health service delivery? What efforts have been made by your group to see to the correction of data discrepancies or to improve the quality of data collected in the health centres? Is the research group operational? 2. Have you reviewed documents and done a literature review for the topic chosen? Have you developed research proposal on areas of mutual interest? 3. Have you engaged in an exercise to support researchers and research resource centres?   MCH     1. Did you assess the personnel trained for the collection of maternal and child health data in the state? 2. How were the findings from the assessment disseminated to the public? 3. Did you carry out advocacy visits to help strengthen data collection for maternal and child health services in the state? Were policy briefs presented to key stake holders in the State? | ****Always ask reason(s) if activity was not executed***  *Give an instance of what it has helped you with. Specifying the aspect*  *Explain in details*  *Give examples.*  *Did you share any data or information with them to help inform policy?*  *Probe for the location of these labs and their strength in terms of skilled human resources and the availability of equipment for appropriate diagnosis.*  *Probe for the stakeholders met, were they adequate, relevant*, *interested, their level of power and the outcome.*    *(Probe for the planning process, implementation of the evaluation program)*  *Probe for the extent they’ve gone with it, the process they’re using for this collection.*  *Probe for the details.*  *Probe for how the documents were reviewed. The extend they have gone with the proposal development and the plans on ground for completion.*  *(Give details of the exercise. Probe for the research centres and the activities they carry out)*  *(Probe for the details of the assessment: planning process, cadre of staff involved, what was done)*  *Give details of the whole exercise.*  *Probe for the stakeholders met, their interests and power levels. The details of the policy brief presentation.* |

**IDI Guide for Evaluation of Proposed GRIPP and HPSR Activities in Anambra State - Users of Evidence**

| **Questions in activity** *(*Note that all proposed respondents are to attempt all questions irrespective of his/her thematic area)* | **Prompts** |
| --- | --- |
| **General**  Generally, how have you used the knowledge gained from the workshop to inform policy or and practice in your office and/ or in your organization/institution at large? (***note in the any of the four thematic area***)  Was there network alliance or any form of engagement created with **producers of evidence** since the last workshop? What was the engagement for and what was the outcome?  **NTDs**   1. Did you build capacity of other stakeholders/health workers working on NTD control in the state/LGs on Evidence Informed Decision Making? If yes could you please tell us how you did that? 2. Which LGA(s) is mostly affected by NTDs and what type is endemic in those areas and. How much drugs were been distributed in those LGA in 2016? What did you use the evidence for? 3. What awareness campaign(s) for NTDs have you or and your group done on NTDs and which channel did you use for the awareness campaign? 4. Are there any advocacy/awareness created or and networking on EBDM you have done since the workshop ended? 5. What facilitated the activities you have been able to carry out since the workshops and what challenges did you encounter?   **Malaria**   1. What HPSR and GRIPP Networking have you or your group establishes or engages in within and outside your unit/organization since the last workshop? What is the outcome of the engagement? 2. Which health research priority did (or any your group) identify? What process(s) where taken and who were the actors involved in the identification? 3. Did you write a memos/proposal to set up or rehabilitate research unit within PRS dept.? Has the department been revived? What strategies were adopted and would you say that the research unit is now functional? If yes why? 4. Did you build capacity and or sensitization on EIDM for any group of people (e.g. program managers) or other health workers? 5. What strategy (s) did you put in place to ensure appropriate documentation of every health intervention done in the SMOH? Was it approved? 6. What facilitated the activities you have been able to carry out since the workshops and what challenges did you encounter?   MCH     1. Did you do step down capacity building for PRS and M&E personnel on Evidence Informed Decision Making (EIDM) in SPHCDA and or SMOH? If yes could you please tell us how? 2. What needs assessment data on malnutrition and or childhood illness did you do? What was the finding(s) and how did you use it to inform practice? 3. What pilot study on M&E of an implemented programme did you conduct? How did you use data obtained to influence immediate decisions in your office for childhood illness such as malaria, diarrhoea or immunization? 4. Was there any health education intervention you and your group conducted for mothers? If yes: how did you achieve that? 5. Did you and or your group carry out a study on why mothers fail to access health care services? How did you do that and what was the finding(s). How did you use it to inform practice or bring about change? 6. What facilitated the activities you have been able to carry out since the workshops and what challenges did you encounter?   HSS   1. Was the research unit in the PRS in the SMOH rehabilitated? Did you write a memos/proposal to set up or rehabilitate research unit within PRS dept.? Has the department been revived? What strategies were adopted and would you say that the research unit is now functional? If yes? Why? 2. Were there Evidence based advocacy/debriefing visit to the commissioner of health/permanent secretary health by you (and or your group) on the outcome and deliberations of the workshop and the need to adopt all the recommendations/potential strategies for EIDM for endemic diseases control and health sector at large? 3. Are there any other people or group of people you and or your group sensitized on EIDM after the last workshop? 4. Did you ensure that the M&E Officers carry out appropriate evaluation of health programmes or interventions? If yes how? 5. What facilitated the activities you have been able to carry out since the workshops and what challenges did you encounter? | ****Always ask reason(s) if activity not executed***  *(Prompts: in reviewing, re-designing programmes, implementation and evaluation plans as well as in any health program budgeting*  (*Explore the following:* *planning and implementation process, and the outcome )*  ****Always ask reason(s) if activity not executed***  *(Explore Processes/methods of data extraction, how/where were data collected from, data analysis method/tool )*  *(Probe for planning process content of the awareness material, channel of communication)*  *(Probe for who, how, where and outcome of the advocacy )*  *(Probe for planning process, content, any established channel of communication, actors level of involved)*   - *(same as in above )* - *(Explore the following: all planning process, actors involved in setting up, state of the department)*   (*Explore the following:* *planning process, implementation of the capacity building, outcome)*  *(Probe for the processes: people involved, for who, how and any form of enforcement or penalty for defaulters)*   - *(Probe for the planning process, actors involved, what was done, current state of the department)*   (*Explore the following:* how *planning process, people involved, content, outcome)*  *(Probes: planning process: who, what was done and how, outcome )*  (*Explore: planning process implementation process, and the outcome )*  *(Explore: planning steps Processes/methods of data collection and extraction, how/where data were collected from, data analysis method/tool ,outcome)*  *(Probe for processes employed where, content, actors and outcome)* |

**IDI Guide for Evaluation of Proposed GRIPP and HPSR Activities in Anambra State - Producers of Evidence**

| **Questions in activity** *(*Note that all proposed respondents are to attempt all questions irrespective of his/her thematic area)* | **Prompts** |
| --- | --- |
| **General**  Generally, how have you used the knowledge gained from the workshop to inform policy or and practice in your office and/ or in your organization/institution at large? (***note in the any of the four thematic area***)  Was there network alliance or any form of engagement created with **users of evidence** since the last workshop? What was the engagement for and what was the outcome?  **NTDs**   1. What steps have you taken to review the existing literature with a view to determining the prevalence of NTDs in the state? 2. What advocacy activities have you carried out with stakeholders EIDM for NTDs in the state since the workshop? 3. What activities, if any have you conducted towards conducting baseline epidemiological studies for NTDs? 4. Are there any other NTD activities that you have carried out or have been involved in since the end of the workshop? 5. What facilitated the activities you have been able to carry out since the workshops and what challenges did you encounter?   **Malaria**   1. What activities have you carried out towards the designing and conducting the Survey “To determine the availability of malaria diagnostic tools and personnel in the LGAs”? 2. What facilitated the activities you have been able to carry out since the workshops and what challenges did you encounter?   **MCH**   1. What activities have you carried out to create awareness and sensitize relevant stakeholders in the state on MCH programmes? 2. What knowledge networks have you set up, if any? 3. What activities have you carried out towards conducting the study on “Uptake of Immunization and/or use of ITNs by pregnant mothers”? 4. What facilitated the activities you have been able to carry out since the workshops and what challenges did you encounter?   **HSS**   1. What activities have you carried out towards Training health workers in NAUTH on “Prompt Attendance to Patients to reduce hospital waiting time”? 2. What steps have been taken towards paying an advocacy visit to the SMoH on “Continuous Sensitization on Evidence Informed Decision Making”? 3. What facilitated the activities you have been able to carry out since the workshops and what challenges did you encounter? | ****Always ask reason(s) if activity not executed***  *(Prompts: in reviewing, re-designing programmes, implementation and evaluation plans as well as in any health program budgeting )*  (*Explore the following:* *planning and implementation process, and the outcome )*  ****Always ask reason(s) if activity not executed***  *(Probe for planning process content of the awareness material, channel of communication)*  *(Probe for who, how, where and outcome of the advocacy*  *(Probe for planning process, content, any channel of communication, actors level of involved) Probe for literature review, writing of study protocol, any networking activities to facilitate process*   - *(Probe for the planning process, actors involved, what was done, current state of the department)*   (*Explore the following:* how *planning process, people involved, content, outcome)*  (*Explore: planning process implementation process, and the outcome )*  *(Probe for processes employed where, content, actors and outcome)* |
